# The impact of surgical technique on the number of sentinel lymph nodes removed and its effect on complication rates

**DOI:** 10.1007/s10549-024-07598-y

**Published:** 2025-01-08

**Authors:** Kathleen Stutz, Holly Mason, Shiva Niakan, Aixa Perez Coulter, Jesse Casaubon, Ann-Kristin U. Friedrich

**Affiliations:** 1 Department of Surgery, Loma Linda University Health, Loma Linda, CA 923754 USA; 2 Sutter Gould Medical Foundation - Breast Health and Gynecology, Modesto, CA 95354 USA; 3 Department of Surgery, University of Massachusetts Chan Medical School - Baystate, Springfield, MA 01199 USA; 4 Office of Research, Epidemiology/Biostatistics Research Core, Baystate Medical Center, Springfield, MA 01199 USA

**Keywords:** Breast cancer, Sentinel lymph node biopsy, Lymph nodes, Breast massage, Complications, Lymphedema

## Abstract

**Purpose:**

Sentinel lymph node biopsy (SLNB) is a staging procedure used to guide treatment for patients with breast cancer. Multiple variations in the SLNB technique have been described. We questioned how technique impacts the number of sentinel lymph nodes (SLNs) removed and associated complications.

**Methods:**

Patients with breast cancer who were treated with lumpectomy and SLNB between 2018 and 2023 were analyzed. Patients were excluded if they had prior ipsilateral breast or axillary surgery or chest wall radiation, underwent neoadjuvant chemotherapy or endocrine therapy, or subsequently required ALND. Demographics, surgical technique, and operative and pathological data were collected. Complication rates were compared between more (4+) or fewer (1–3) SLNs removed.

**Results:**

A total of 643 patients were included, with an average of 2.44 LNs removed (range 1–11). The overall complication rate was 19.8%, with a 4.4% lymphedema rate. The lymphedema rate was higher among patients who had more nodes removed. An average of 2.5 LNs were removed with dual mapping vs. 2.0 with technetium alone (p = 0.15). Breast massage had no effect on the number of SLNs removed (p = 0.12) but did impact blue dye uptake (p = 0.001).

**Conclusions:**

Surgical technique did not significantly impact the number of nodes removed. Removing more nodes was associated with a greater risk of lymphedema.

## Introduction

Lymph node (LN) metastasis is the most important predictor of recurrence (locoregional and distant) and overall survival in patients with breast cancer [1, 2]. Patients who are newly diagnosed with breast cancer undergo axillary examination, which includes physical examination and often imaging studies such as ultrasound or MRI [3]. SLNB is routinely performed at the time of breast cancer surgery to allow for axillary staging in clinically node negative patients.

SLNB is based on the hypothesis originally proposed by Cabanas [4]. He proposed that tumor cells shed from a primary carcinoma and migrate through a lymphatic channel to a single lymph node before involving further lymph nodes within that basin. The sentinel lymph node, therefore, is the first lymph node that receives lymphatic drainage from a tumor, and its identification and analysis for tumor involvement should predict the status of the remaining lymph nodes [5]. SLNB involves mapping the lymphatic drainage from the breast via dyes to identify the first lymph node, or group of lymph nodes, that drain the breast and help to determine if the cancer has begun to spread. SLNB removes an average of 1–4 lymph nodes, whereas axillary lymph node dissection (ALND) involves removal of all the lymph nodes in the axilla within certain anatomic borders (the axillary vein superiorly, pectoralis major muscle anteriorly, thoracodorsal neurovascular bundle posteriorly, axillary skin laterally, and the chest wall and long thoracic neurovascular bundle medially). ALND should remove 10 or more lymph nodes [7]. SLNB has replaced ALND due to the increased risk for postoperative complications while still allowing for axillary staging. The most significant complication associated with ALND is lymphedema of the arm, which can lead to additional sequelae, such as decreased arm function or even subsequent malignancy [6].

Other complications associated with axillary lymph node removal include seroma, hematoma, cellulitis, abscess, nerve injury, arm stiffness and axillary cording. Multiple studies have compared the morbidity of ALND and SLNB. One meta-analysis that included 7415 articles reported increased rates of lymphedema (16.5–23.6% after ALND vs 5.9–7.5% after SLNB) and long-term pain scores; a greater proportion of patients experienced a reduced range of motion (29.8% ALND vs 17.1% SLNB), reduced strength and upper limb function, and decreased quality of life after ALND [6, 8]. ALND can even lead to additional malignancies [9]. Furthermore, removing additional lymph nodes has not been found to improve overall patient survival [10–13]. Instead, the greater the number of lymph nodes that are removed, the greater the likelihood of complications and the poorer the quality of life [14, 15]. The call for the de-escalation of axillary surgery is motivated by the desire to reduce upper extremity morbidity while maintaining locoregional control and overall survival [16].

At our institution, the SLNB procedure typically involves the preoperative injection of a radioactive dye (Technetium-99 or LymphoSeek), allowing the surgeon to use a gamma counter-like probe to identify the sentinel lymph node(s). Most surgeons also inject a colored dye, typically isosulfan blue or methylene blue, that stains the lymph nodes for visual identification. After the dyes are injected, some surgeons choose to massage the breast. The purpose of breast massage is to promote dye entry into the lymphatic channels and enhance travel to the lymph nodes [17]. There is no standard procedure regarding how much dye is injected, whether massage is performed, or its duration or technique.

Evaluating the variation in surgical technique may identify consistent factors associated with the identification of a greater number of sentinel nodes and/or increased number of resulting complications. We hypothesize that performing breast massage can lead to improved uptake of dyes within the axilla, allowing the identification and removal of a greater number of sentinel lymph nodes. Some studies cite a lower false negative rate with removal of more lymph nodes, however there is concern for overtreatment and the removal of additional nodes that are not actually sentinel [15, 18, 19]. Moreover, we know that the removal of a greater number of lymph nodes correlates with higher complication rates and poorer quality of life [14, 15].

## Methods

We conducted a retrospective review of women aged 18 years and older with new diagnosis of breast cancer who underwent lumpectomy and sentinel lymph node biopsy at our regional medical center as their first treatment between January 1, 2018, and January 1, 2023. The study was evaluated by the Institutional Review Board and deemed exempt. Data were collected via the institution’s IRB-approved Breast Disease Patient Repository, a secure, HIPAA-compliant REDCap database that is prospectively maintained (grant number UL1TR002544). The records were extracted from the institutional electronic medical records. Patients were excluded if they underwent mastectomy; had prior ipsilateral lymph node surgery; had ipsilateral breast, axillary, or chest wall radiation; had undergone neoadjuvant chemotherapy or neoadjuvant endocrine therapy; or had undergone subsequent ALND.

Our primary endpoint was the number of lymph nodes removed during the SLNB procedure. Secondary endpoints included the rate of local (axillary) complications after the SLNB procedure.

Data were collected on patient demographics (age, sex, reported race, ethnicity), tumor data and receptor status, operating surgeon, amount and location of blue dye injection, whether or not technetium was injected, whether or not massage was performed, whether or not technetium and/or blue dye was identified in the axilla, the timing of the sentinel node biopsy procedure (on the same day as lumpectomy or delayed), the number of sentinel lymph nodes removed, and complications. Data were collected on the pathologic status of the retrieved lymph nodes. Treatment details were also recorded. The data were grouped and compared on the basis of the number of lymph nodes removed (fewer lymph nodes (1–3) vs more (4+) lymph nodes).

We initially evaluated differences in patient characteristics across study groups. All continuous variables were checked for normality via skewness and kurtosis statistics. If a continuous variable had a skewness or kurtosis statistic above an absolute value of 2.0, then the variable was assumed to have a nonnormal distribution. The results for continuous variables are shown as either the mean and standard deviation (SD) for normally distributed variables or the median and interquartile range (IQR) for skewed variables, whereas categorical variables are shown as n and %. A t-test was used for continuous variables with a normal distribution, and the Mann‒Whitney U test was used for skewed variables. The chi-square test and Fisher’s exact test were used for categorical variables. A Spearman's correlation was run to assess the relationship between age and number of lymph nodes removed.

## Results

### Patient demographics

A total of 643 women met the inclusion criteria; the median age was 59.9 (50.4–69.4) years. Most patients were Caucasian (67.9%), had invasive ductal carcinoma (80.2%), and had estrogen receptor positive (88%), HER2 negative (84.6%) breast cancer (Table 1).

**Table 1:** Demographics

n (%)	643 (100.0)
Age at diagnosis, mean (sd)	59.9 (9.3)
Race/ethnicity, n (%)	
White/Caucasian	436 (67.9)
Black/AA	28 (4.4)
Hispanic/Latino	51 (7.9)
Asian/Pacific Islander	15 (2.3)
Native American/Alaska Native/Indian	1 (0.2)
Other/unknown	111 (17.3)
Ethnicity, n (%)	
Hispanic	51 (7.9)
Non-Hispanic	483 (75.1)
Unknown	109 (17.0)
Tumor type, n (%)	
DCIS	27 (4.2)
Invasive ductal	516 (80.2)
Invasive lobular	61 (9.5)
Other	39 (6.1)
Tumor grade, n (%)	
1	268 (41.7)
2	252 (39.2)
3	121 (18.8)
Unknown	2 (0.3)
ER status, n (%)	
No	76 (11.8)
Yes	566 (88.0)
Unknown	1 (0.2)
PR status, n (%)	
No	135 (21.0)
Yes	505 (78.5)
Unknown	3 (0.5)
HER-2 positivity, n (%)	
No	544 (84.6)
Yes	45 (7.0)
N/A	51 (7.9)
Equivocal/unknown	3 (0.4)

There was a weak negative correlation between age and number of lymph nodes removed, which was statistically significant, r_s_ = − 0.0796, p = 0.0437 (p-value < 0.05), indicating that older age was associated with a lesser number of lymph nodes removed.

### Technique

A total of 643 sentinel lymph node biopsy procedures were performed by seven different surgeons. The range of sentinel lymph nodes removed was 1–11, with an average of 2.44 lymph nodes excised.

**Fig. 1:**
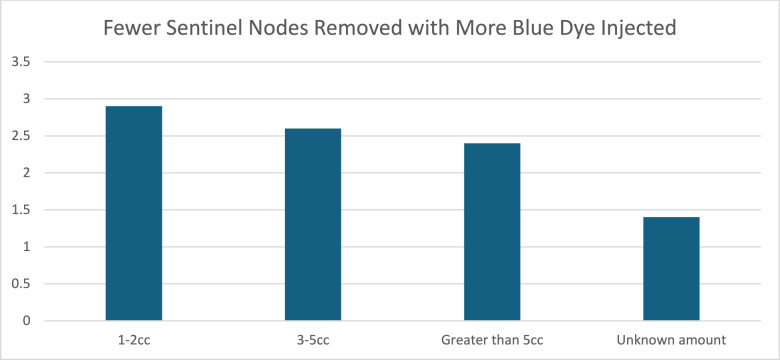
Fewer sentinel nodes removed with more blue dye injected

**Fig. 2:**
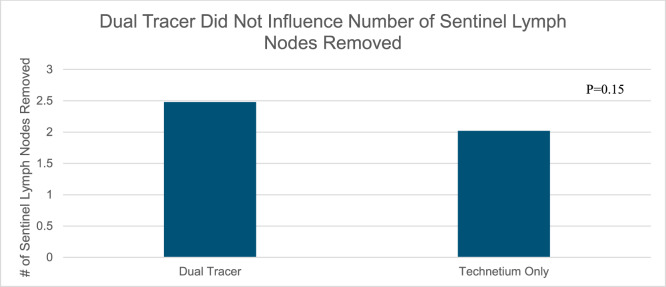
Dual tracer did not influence number of sentinel lymph nodes removed

Dual tracer (isosulfan or methylene blue dye and technetium-99) was injected into 585 (90.9%) patients, whereas 50 patients (7.7%) received only technetium-99. When blue dye was used, 3–5 cc of dilute blue dye was most commonly injected in a periareolar or subareolar fashion (79.7%), and one patient (0.15%) received a peritumoral injection of dye. In 8 patients (1.2%), the injection location was not documented. A higher volume of blue dye injection correlated with a lower number of sentinel nodes removed (Table 2; Fig. 1). The number of removed lymph nodes was slightly greater on average when dual tracer was used (2.5 vs 2.0); however, this difference was not statistically significant (p = 0.15, Fig. 2).

**Table 2:** Higher volume of blue dye injection results in less sentinel lymph nodes removed (P = 0.02)

	1–2 cc	3–5 cc	Greater than 5 cc	Unknown amount
N (%)	39 (6.7)	391 (66.8)	14 (2.4)	141 (24.1)
Number of sentinel nodes removed, mean (SD)	2.9 (2.0)	2.6 (1.7)	2.4 (1.2)	2.1 (1.4)

**Fig. 3:**
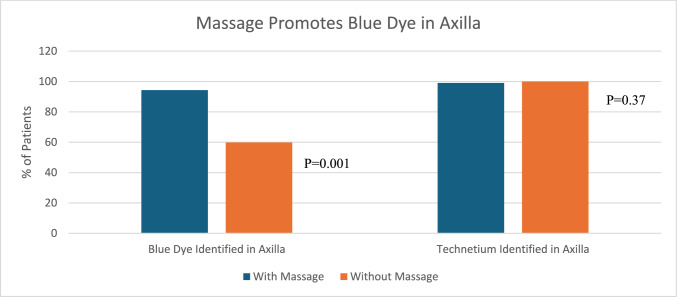
Massage promotes blue dye in the axilla

**Fig. 4:**
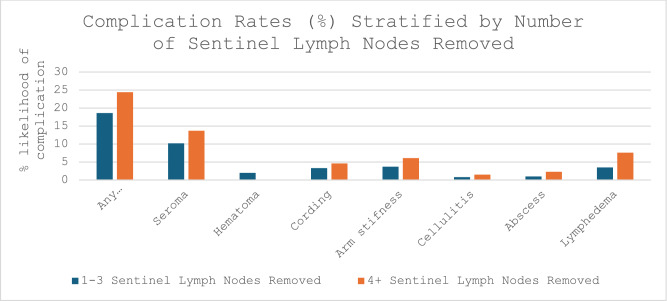
Complication rates (%) stratified by the number of sentinel lymph nodes removed

Breast massage after injection of the tracer was performed in 418 (83.6%) patients. Massage was associated with greater uptake of blue dye in the axilla (94.3% with massage vs 59.8% without massage, p = 0.001). Breast massage did not show an association with the overall number of sentinel lymph nodes removed (2.4 nodes with massage vs. 2.6 nodes without, p = 0.12) (Figs. 3, 4).

### Complications

The overall complication rate was 19.8%, with a 4.4% lymphedema rate. Complication rates were compared between patients who had 1–3 lymph nodes removed and those who had 4 + lymph nodes removed.

The probability that a patient would experience any complication after 1–3 lymph nodes were removed was 18.6% compared with 24.4% of patients who had 4 + lymph nodes removed. There was a higher lymphedema rate among patients who had more nodes removed (7.6% with 4 + nodes removed vs 3.5% with 1–3 nodes removed, p = 0.04). Other axillary complications included cellulitis, postoperative abscess, seroma, hematoma, cording, and arm stiffness. Complication rates were similar between the two groups. Cellulitis was experienced by 0.8% of patients who had 1–3 lymph nodes removed vs. 1.5% of patients who had 4 LNs removed (p = 0.43). All patients received antibiotics for treatment of their cellulitis. Postoperative abscesses were observed in 1.0% and 2.3% of the patients, respectively (p = 0.23). A seroma was noted in 10.2% of patients who had 1–3 lymph nodes removed, whereas it was noted in 13.7% of patients who had 4 + lymph nodes removed (p = 0.24). Most patients with seroma required aspiration (68.6%), and of these, 6.3% required a seroma catheter.

Hematomas were rare and were encountered in 2.5% of patients who had 1–3 lymph nodes removed. Among patients who had 4 + lymph nodes removed, no hematomas occurred (p = 0.11). Most cases (6 patients) were managed conservatively, while 3 patients required aspiration, and one patient returned to the operating room for hematoma evacuation. 6 patients had no documentation regarding management of their hematoma.

Similar incidences of axillary cording (3.3% of patients who had 1–3 LNs removed vs 4.6% for those who had 4 + LNs removed, p = 0.49) and arm stiffness (3.7% of patients who had 1–3 LNs removed vs 6.1% of patients with 4 + lymph nodes excised, p = 0.22) were noted when comparing the two groups. Although our data demonstrated a trend toward increased complication rates with a greater number of lymph nodes removed, there was no statistically significant difference between the groups for any of the complications other than lymphedema (Table 3).

**Table 3:** Complication rates (%) stratified by the number of sentinel lymph nodes removed

	1–3 LNs removed	4 + LNs removed	Overall/total	P value
Any complication	18.6	24.4	19.8	0.13
Seroma	10.2	13.7	10.9	0.24
Hematoma	2	0	1.6	0.11
Cording	3.3	4.6	3.6	0.49
Arm stiffness	3.7	6.1	4.2	0.22
Cellulitis	0.8	1.5	0.9	0.43
Abscess	1	2.3	1.2	0.23
Lymphedema	3.5	7.6	4.4	0.04

## Discussion

Axillary surgery can be followed by debilitating lymphedema and decreased range of motion, both of which can significantly impact quality of life [9]. Multidisciplinary care teams are placing increasing emphasis on the diagnostic rather than therapeutic purpose of axillary surgery. Recent trials have aimed to identify alternative therapies to decrease and, at times, exclude axillary surgery altogether [20–24]. It is well known that removing a greater number of lymph nodes places the patient at higher risk for axillary complications [14]. Furthermore, removing additional lymph nodes has not been found to improve overall patient survival [12, 13]. To our knowledge, there are no studies aimed at evaluating the technique of sentinel lymph node biopsy with respect to dye injection, breast massage and its impact on the number of sentinel lymph nodes removed.

Our study explores the preprocedural steps of sentinel lymph node biopsy to identify methods that may increase the number of sentinel lymph nodes removed, which could thereby increase complication rates without simultaneous diagnostic benefit. In a meta-analysis by Kim et al., the average number of sentinel lymph nodes excised during SLNB was 1.92 and ranged from 1 to 4.1 sentinel lymph nodes across studies [25]. Our study revealed a slightly greater number of sentinel lymph nodes excised.

The intraoperative identification of sentinel lymph nodes in patients with breast carcinoma was shown to be successful by Krag et al. using technetium-99, Giuliano et al. using blue dye and Albertini et al. using a combination of technetium and blue dye with initial identification rates reported of 82%, 66%, and 92% respectively [26–28]. Subsequent studies have assessed uptake of radiotracer alone, blue dye alone and dual tracer. When radioisotope tracer alone is used, uptake and identification range from 85.6 to 97.6% [29]. Uptake of blue dye in the axilla when used alone for identification of the sentinel lymph node, ranges widely from 16.7 to 97.4% [30–32]. The use of dual tracer approaches a 95–100% identification rate [32].

The purpose of breast massage is to promote dye entry into the lymphatic channels and enhance travel to the lymph nodes [17]. Our study demonstrated an 81% uptake of blue dye in the axilla with breast massage and 56.3% uptake without massage. Given the wide range reported in the studies noted above, this is consistent with published data. However, it should be noted that in 69 (14%) patients who received breast massage and 5 (5.7%) patients who did not receive breast massage, identification of blue dye within the axilla was not included within the operative report and therefore data could not be reported for these patients. Excluding the patients with missing data, blue dye was identified in the axilla in 396 out of 420 patients (94.3%) who had breast massage and 49 out of 82 (59.8%) of patients who did not receive breast massage. Since massage did not appear to have a significant impact on finding technetium in the axilla but did influence uptake of blue dye in the axilla, one may infer that performing a breast massage may be helpful when performing a sentinel lymph node biopsy with blue dye only.

Performance of breast massage after blue dye injection increases the propensity of finding blue dye within the axilla but does not increase the overall number of sentinel lymph nodes removed. Interestingly, we found that the volume of blue dye injected was inversely related to the number of sentinel lymph nodes identified and removed. This was an unexpected finding of our dataset. We hypothesize that there may have been additional blue dye injected in patients when poor technetium uptake was noticed by the operating surgeon, indicating an overall problem with lymphatic mapping for certain patients, but were unable to evaluate this due to lack of details in the operative documentation. Moreover, there is a paucity of data in the current literature correlating volume of blue dye injected to number of sentinel lymph nodes removed.

Although there was a trend toward an increased number of sentinel nodes removed with the use of dual tracers (2.5 vs 2.0 lymph nodes), this difference was not statistically significant. However, our study may have been underpowered to demonstrate this difference.

A false negative rate (FNR) of less than 10% has been deemed the acceptable and expected rate for sentinel lymph node surgery in women who initially present with clinically negative axillary lymph nodes [24, 34, 35]. The NSABP B-32 trial demonstrated a decrease in the FNR as more sentinel lymph nodes were resected: 18%, 10% and 7% with 1, 2 and 3 SLNs resected, respectively [36]. The American College of Surgeons Oncology Group (ACOSOG) Z1071 Clinical Trial reported a decreased false negative rate (FNR) when dual tracer consisting of a blue dye plus a radiolabeled colloid mapping agent was used and when 3 or more SLNs were removed after neoadjuvant chemotherapy (NAC) [24]. Similarly, Hunt et al. showed that removal of fewer than 2 SLNs was associated with a higher FNR in patients with clinically node-negative disease who underwent SLN surgery after chemotherapy [37].

Krag et al. hypothesized that, to lower the FNR, the surgeon might be motivated to remove more rather than fewer nodes [36]. Multiple studies have demonstrated that an increased number of lymph nodes are removed with the use of dual-tracer over a single-tracer technique [30–32, 38]. However, we must be vigilant and strive to balance the specificity of sentinel lymph node biopsy with known complications as more lymph nodes are excised. Although not statistically significant, our data demonstrated a propensity to remove a greater number of lymph nodes (2.5) when dual tracer was used over single tracer (2.0 lymph nodes). Considering our findings and previously published data we consider it plausable that the use of dual tracer or breast massage could lead to an unnecessary increase in the number of lymph nodes removed, may not be necessary to increase the success rate of SLNB for non-NAC patients and could contribute to overtreatment.

Our study similarly revealed the previously well-known finding that a greater number of axillary lymph nodes removed corresponds to increased rates of lymphedema (7.6% with 4 + nodes removed vs 3.5% with 1–3 nodes removed, p = 0.04) and demonstrated a propensity for increased overall complications, although this difference was not statistically significant.

Limitations of our study include its retrospective nature and limited consistency in the reporting of preprocedural techniques. This variation in reporting likely affected the power of our study and thereby lack of statistical significance. This appears to be a common concern among other previously published studies [25]. Our study may also have been underpowered to detect other subtle differences and correlations between surgical technique and lymph node removal.

## Conclusion

The findings of this study suggest that breast massage increases the uptake of blue dye within the axilla. However, our study did not demonstrate that nuances in surgical technique, including the use of blue dye or breast massage, affected the number of lymph nodes removed. Interestingly, we demonstrated a negative correlation between volume of blue dye injected and number of lymph nodes removed; this highlights a gap in current literature that would benefit from further investigation. Lastly, this study supports prior findings that removing fewer lymph nodes is associated with a lower risk of lymphedema.

## Data Availability

Data was collected using the institution’s IRB-approved Breast Disease Patient Repository, a secure, HIPAA compliant REDCap database which is prospectively maintained (grant number UL1TR002544). Record abstractions were gathered from the institutional electronic medical record. The REDCap database and electronic medical records are kept private and are not publicly available in order to protect study participant privacy.
